# Anthropogenic uranium signatures in turtles, tortoises, and sea turtles from nuclear sites

**DOI:** 10.1093/pnasnexus/pgad241

**Published:** 2023-08-22

**Authors:** Cyler Conrad, Jeremy Inglis, Allison Wende, Matthew Sanborn, Nilesh Mukundan, Allison Price, Travis Tenner, Kimberly Wurth, Benjamin Naes, Jeanne Fair, Earl Middlebrook, Shannon Gaukler, Jeffrey Whicker, Jamie L Gerard, Washington Tapia Aguilera, James P Gibbs, Blair Wolf, Tonie K Kattil-deBrum, Molly Hagemann, Jeffrey A Seminoff, Timothy Brys, Rafe Brown, Katrina M Derieg

**Affiliations:** Earth Systems Science Division, Risk and Environmental Assessment Group, Pacific Northwest National Laboratory, P.O. Box 999, Richland, WA 99352, USA; Department of Anthropology, University of New Mexico, Albuquerque, NM 87131, USA; Chemistry Division, Nuclear and Radiochemistry Group, Los Alamos National Laboratory, P.O. Box 1663, Los Alamos, NM 87545, USA; Chemistry Division, Nuclear and Radiochemistry Group, Los Alamos National Laboratory, P.O. Box 1663, Los Alamos, NM 87545, USA; Chemistry Division, Nuclear and Radiochemistry Group, Los Alamos National Laboratory, P.O. Box 1663, Los Alamos, NM 87545, USA; Chemistry Division, Nuclear and Radiochemistry Group, Los Alamos National Laboratory, P.O. Box 1663, Los Alamos, NM 87545, USA; Chemistry Division, Nuclear and Radiochemistry Group, Los Alamos National Laboratory, P.O. Box 1663, Los Alamos, NM 87545, USA; Chemistry Division, Nuclear and Radiochemistry Group, Los Alamos National Laboratory, P.O. Box 1663, Los Alamos, NM 87545, USA; Chemistry Division, Nuclear and Radiochemistry Group, Los Alamos National Laboratory, P.O. Box 1663, Los Alamos, NM 87545, USA; Chemistry Division, Nuclear and Radiochemistry Group, Los Alamos National Laboratory, P.O. Box 1663, Los Alamos, NM 87545, USA; Bioscience Division, Genomics and Bioanalytics Group, Los Alamos National Laboratory, P.O. Box 1663, Los Alamos, NM 87545, USA; Bioscience Division, Genomics and Bioanalytics Group, Los Alamos National Laboratory, P.O. Box 1663, Los Alamos, NM 87545, USA; Environmental Protection and Compliance Division, Environmental Stewardship Group, Los Alamos National Laboratory, P.O. Box 1663, Los Alamos, NM 87545, USA; Environmental Protection and Compliance Division, Environmental Stewardship Group, Los Alamos National Laboratory, P.O. Box 1663, Los Alamos, NM 87545, USA; Environmental Protection and Compliance Division, Environmental Stewardship Group, Los Alamos National Laboratory, P.O. Box 1663, Los Alamos, NM 87545, USA; Galápagos Conservancy, 11150 Fairfax Blvd. #408, Fairfax, VA 22030, USA; University of Málaga, Campus Teatinos, Apdo 59.29080 Málaga, Spain; Galápagos Conservancy, 11150 Fairfax Blvd. #408, Fairfax, VA 22030, USA; College of Environmental Science, State University of New York, 1 Forestry Dr., Syracuse, NY 13210, USA; Department of Biology, University of New Mexico, Albuquerque, NM 87131, USA; Burke Museum, University of Washington, 4300 15th Ave. NE, Seattle, WA 98105, USA; Department of Natural Sciences, Vertebrate Zoology, Bishop Museum, 1525 Bernice Street, Honolulu, HI 96817, USA; NOAA-Southwest Fisheries Science Center, 8901 La Jolla Shores Drive, La Jolla, CA 92037, USA; Community Engagement and Collections Management, Perot Museum of Nature and Science, Dallas, TX 75201, USA; Natural History Museum and Biodiversity Institute, University of Kansas, Dyche Hall, 1345 Jayhawk Blvd., Lawrence, KS 66045, USA; Natural History Museum of Utah, University of Utah, 301 Wakara Way, Salt Lake City, UT 84108, USA

**Keywords:** chelonian, radionuclide, uranium, plutonium, nuclear fallout

## Abstract

Chelonians (turtles, tortoises, and sea turtles) grow scute keratin in sequential layers over time. Once formed, scute keratin acts as an inert reservoir of environmental information. For chelonians inhabiting areas with legacy or modern nuclear activities, their scute has the potential to act as a time-stamped record of radionuclide contamination in the environment. Here, we measure bulk (i.e. homogenized scute) and sequential samples of chelonian scute from the Republic of the Marshall Islands and throughout the United States of America, including at the Barry M. Goldwater Air Force Range, southwestern Utah, the Savannah River Site, and the Oak Ridge Reservation. We identify legacy uranium (^235^U and ^236^U) contamination in bulk and sequential chelonian scute that matches known nuclear histories at these locations during the 20th century. Our results confirm that chelonians bioaccumulate uranium radionuclides and do so sequentially over time. This technique provides both a time series approach for reconstructing nuclear histories from significant past and present contexts throughout the world and the ability to use chelonians for long-term environmental monitoring programs (e.g. sea turtles at Enewetok and Bikini Atolls in the Republic of the Marshall Islands and in Japan near the Fukushima Daiichi reactors).

**Significance Statement**

Chelonians (turtles, tortoises, and sea turtles) bioaccumulate anthropogenic radionuclides (e.g. ^235^U and ^236^U) in their shell scute keratin. Since chelonian scute grows sequentially over time, this approach provides the ability to reconstruct nuclear fallout or contamination histories and conduct environmental monitoring, in localized settings. We identify uranium signatures relating to historic nuclear testing or accidental release in a green sea turtle from the Republic of the Marshall Islands, a desert tortoise from southwestern Utah (United States of America), a river cooter from the Savannah River Site, and a box turtle from the Oak Ridge Reservation.

## Introduction

In a 1952 United States Civil Defense Administration educational film titled “Duck and Cover”, an animated turtle named “Bert” showed the American public what to do in case of a nuclear attack—the words were simple, you should “duck” and “cover”, and for millions of schoolchildren, this meant seeking shelter under their desks. Shelter for the anthropomorphic Bert involved a functional characteristic present in some chelonian taxa (turtles, tortoises, and sea turtles), the ability to retract his head and feet into his bony shell. Although a fictional depiction, Bert provides a useful analogy for understanding the effects of nuclear activities in the environment today. Effects from nuclear activities leave a legacy of specific elemental and isotopic signals in environments near and far from nuclear sites or detonation locations (1, 2). For anthropogenic uranium radionuclides, they are long-lived, globally distributed, and bioaccumulate within sediments, plants, and animals (3–6). In this study, we measure uranium (^235^U and ^236^U) in a variety of chelonian scute tissue from areas with a known history of contamination from nuclear testing (fallout) and/or nuclear processing (waste). Our research highlights that Bert's fictional experience was in some ways true—turtles, tortoises, and sea turtles live in areas with historic nuclear activity and may have survived the immediate effects of nuclear events—but our study also confirms that the record of this nuclear activity is present and measurable within these same animals long after first exposure.

### Anthropogenic uranium isotopes

Globally, there are numerous potential areas to investigate legacy radionuclide bioaccumulation in chelonians. Throughout the 20th-century Cold War, the United States developed a large nuclear weapons complex, involving multiple sites across the country devoted to producing fissile and other nuclear weapons materials from natural uranium. These early activities produced contamination in the environment, before our understanding of its impact on the environment led to better management practices. In the United States alone, there is an estimated 30–80 million m^3^ of contaminated soil and 1.8–4.7 billion m^3^ of contaminated water from legacy nuclear activities (7). Uranium represents an important component of the contamination associated with these legacies of Cold War nuclear weapons production, as its isotopic composition carries distinct information about the nuclear processes it experienced—and therefore acts as a fingerprint of the activities occurring at a particular site. In addition, these radionuclides are long-lived in the environment (e.g. ^235^U has a half-life of 703.8 million years).

Processing activities for natural uranium (0.7% ^235^U) involved enrichment to produce highly enriched uranium (HEU, >20% ^235^U) for direct use in nuclear weapons or for use in nuclear reactors to produce fissile plutonium. Depleted uranium (DU, <0.7% ^235^U), a by-product of both enrichment and reactor processes, was produced in large quantities and initially found limited use, so was stored or disposed of as waste (it was also used in military munitions). Significant amounts of ^236^U were also produced anthropogenically by thermal neutron activation of ^235^U during nuclear fission, and a lesser amount by the ^238^U(n/3n)^236^U nuclear reaction. When input into the environment, this anthropogenic ^236^U easily overrides the natural ^236^U/^238^U signature (typically in the range of 10^−10^–10^−14^), and provides a clear indicator of contamination through anthropogenic uranium (8). As there are no natural sources of HEU, DU, or ^236^U in the environment, then, identification of such isotopically unique signatures of uranium in plants, soils, and animals provides a fingerprint of nuclear activities in a region. Temporal signatures, in plants or animals that grow sequential tissues, also provide additional fidelity to our understanding of the fate of these effluents in the environment.

**Fig. 1.**
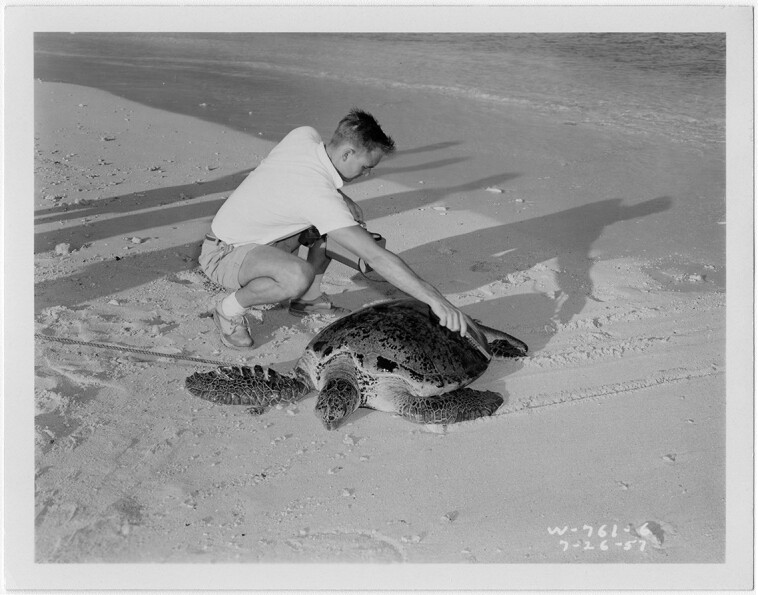
A U.S. Atomic Energy Commission photograph from July 26, 1957 showing an individual using a Geiger counter to examine a green sea turtle (*Chelonia mydas*) for potential radioactivity in the Republic of the Marshall Islands. The location is not provided, but based on historic context and associated photographs in this series, it is likely from Enewetak Atoll. Courtesy of the National Archives, photo no. 28828530 (326-16-042).

Here, we investigate records of anthropogenic radionuclide activity, specifically historic Cold War releases of DU and HEU into the environment, as it relates to 20th-century turtles, tortoises, and sea turtles (Fig. 1) from nuclear sites. Previous bulk bone ^137^Cs and ^90^Sr research on a variety of chelonian taxa indicates that there is potential to use these organisms as records of anthropogenic nuclear activities (9–14). However, unlike previous research that focused on analysis of bulk bone tissue (or whole turtles), or only the anthropogenic accumulation of radiocarbon (15), we show that sequentially grown turtle scute keratin is an inert reservoir that captures signatures of nuclear contamination in chronologically controlled intra-chelonian samples.

### Chelonians at nuclear sites

**Fig. 2.**
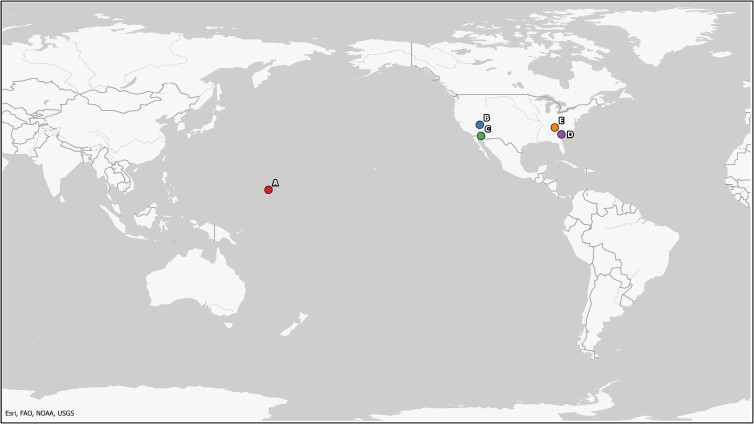
The location of turtles analyzed in this study. A) Enewetak Atoll, Republic of the Marshall Islands, B) southwestern Utah, C) Barry M. Goldwater Air Force Range, D) the Savannah River Site, E) Oak Ridge Reservation. Figure courtesy of Bethann McVicker (Los Alamos National Laboratory GIS Program).

Our analysis focused on chelonian specimens from areas that potentially accumulated anthropogenic uranium through nuclear fallout and/or waste (Fig. 2). For example, turtles from the Republic of the Marshall Islands and near the Nevada National Security Site may have bioaccumulated radionuclides from nuclear test fallout. In contrast, turtles at the Oak Ridge Reservation (or other national laboratory sites) may have bioaccumulated radionuclides from uranium waste products through production and processing activities. We identified five distinct chelonian specimens from natural history collections for analysis and conducted first order measurements on bulk scute keratin to determine presence of anthropogenic uranium. A candidate scute with the potential for temporal examination then provided a second order of analysis to investigate change in anthropogenic uranium compositions over time.

The first bulk specimen is from a green sea turtle (*Chelonia mydas*; [hereafter, museum catalogue number] BPBM-6179) scute collected on 1978 May 1 from the stomach of a tiger shark caught by line fishing off a pier at Enewetak Island, Enewetak Atoll, Republic of the Marshall Islands (RMI). Enewetak Atoll, and neighboring Bikini Atoll, were the site of 67 nuclear weapons tests from 1946–58 (16) that resulted in contamination of both islands and the surrounding lagoons. We believe that this specimen was ∼10–20 years old when collected, based on scute size. Nuclear testing ended 20 years prior to the collection of this sample in 1958. Thus, we hypothesize that it is unlikely that this sea turtle was alive during nuclear testing at Enewetak Atoll in the 1950s.

Green sea turtles migrate, forage, and nest throughout the RMI, and satellite telemetry indicates that adult nesters travel throughout the equatorial regions of the western Pacific Ocean during their lifetimes (17). Although we do not know the exact migratory history of this green turtle specimen, its presence at Enewetak Atoll indicates that it likely foraged within the atoll environment prior to its death.

The second bulk specimen scute is from a Mohave desert tortoise (*Gopherus agassizii*; UMNH:Herp:3571) collected on 1959 April 23 from Washington County, southwestern Utah. This scute is from an old, mature, individual tortoise with homogenized layers (i.e. no layer rings present due to its old age). Washington County is approximately 150 miles due east of the (formerly) Nevada Test Site (now Nevada National Security Site) where the Atomic Energy Commission (now Department of Energy) tested 100 atmospheric nuclear weapons between 1951 and 1962. Radionuclide data indicate that regions of Washington County, southwestern Utah, such as St. George and Cedar City, received low levels of fallout from weapons tests approximately 5–6 times through 1959 (18).

The third bulk specimen scute is from a Sonoran desert tortoise (*Gopherus morafkai*; Department of Biology, University of New Mexico) collected in 1999 at the Barry M. Goldwater Air Force Range (BMGAFR) in southwestern Arizona. Unlike our other samples, this region experienced no direct input of anthropogenic uranium (e.g. no known use of DU munitions) and therefore represents a natural control from an arid environment.

The fourth bulk specimen scute is from a river cooter (*Pseudemys concinna*; KU-204360) collected from the Savannah River Site (SRS), South Carolina in 1985. From the 1950s to the late-1980s, the SRS was an important location of uranium fuel fabrication, target fabrication, and spent fuel reprocessing. A historical inventory of uranium waste disposed at the SRS indicated a total of 198.15 metrics tons present during this period from at least one location at the site, primarily comprised of fuel fabrication and spent fuel processing, including DU (19). The scute from this specimen reflects approximately one year of growth due to the presence of scute shedding in this taxon (20).

The collection history for this river cooter specimen indicates that it was collected from the SRS, but we lack a more specific location from within the site. Today, the SRS is over 198,000 acres (or ∼310 mi^2^) in size. River cooters are aquatic, move easily within aquatic habitats, and may travel short distances by land if necessary (21, 22). Resource availability largely influences their movement patterns over days and seasons. Migration and home range studies from Florida and Illinois (United States of America) show that river cooters move up to hundreds of meters per day, and in rarer cases over a kilometer or more, both of which are on a scale much smaller than the area of the SRS. These movement characteristics support our interpretation that this river cooter lived at the SRS throughout its life, even if the exact location of its collection remains unknown.

The fifth sequential specimen scute is from an eastern box turtle (*Terrapene carolina carolina*; DMNH-3835) collected in 1962 from the Oak Ridge Reservation (ORR). Like the SRS, ORR was the site of significant uranium production and processing beginning in 1943 (23). The site included the K-25 gaseous enrichment plant, which produced a variety of uranium enrichments up to 97% HEU, and the Y-12 Complex, which produced weapons components. Historical waste management records from ORR indicate the release of significant volumes of DU and HEU into the environment surrounding ORR via both airborne and liquid waste streams (23). In addition to a bulk scute measurement, this specimen exhibited clear growth layers encompassing 7 years of life (1955–62), including the presence of the neonatal period of development. This unique layering, in comparison to the scutes analyzed as bulk samples, enabled a diachronic investigation of anthropogenic radionuclide contamination between 1955 and 1962.

## Results

### Bulk scute samples

Using multi-collector inductively coupled plasma mass spectrometry (MC-ICP-MS), we identified a large degree of variability in the uranium isotopic composition across the bulk scutes analyzed, including significant variations in both ^235^U/^238^U and ^236^U/^238^U. We also measured ^234^U/^238^U, but this is not discussed further as ^234^U/^238^U is known to vary widely in nature (24).

**Fig. 3.**
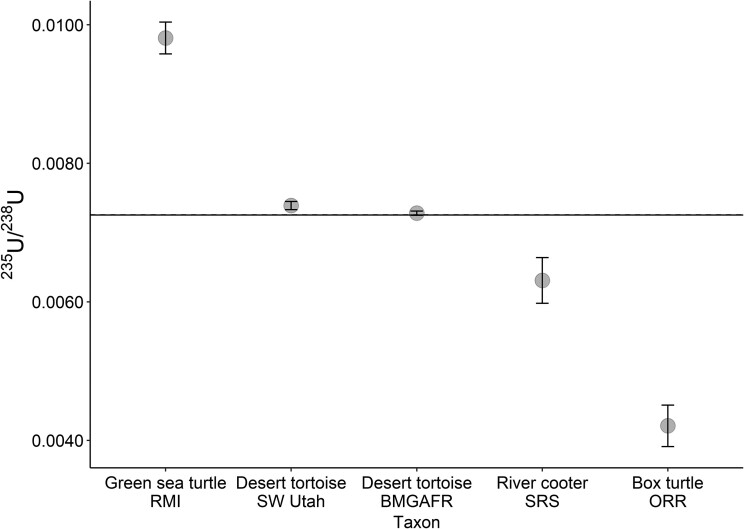
The ^235^U/^238^U isotopic ratio and error for bulk turtle scute tissue samples from this study. Natural uranium (0.007253–0.007256) is visible as a horizontal line. RMI, Enewetak Atoll, Republic of the Marshall Islands; SW Utah, southwestern Utah; BMGAFR, Barry M. Goldwater Air Force Range; SRS, the Savannah River Site; ORR, Oak Ridge Reservation.

In four out of the five samples analyzed, the ^235^U/^238^U deviated from that observed in natural terrestrial materials (25), and included both depleted and enriched signatures (Fig. 3; Table 1). Only the scute from the Sonoran desert tortoise from the BMGAFR had a ^235^U/^238^U ratio that is indistinguishable from natural uranium isotopic compositions. The bulk scute from the area surrounding the uranium fuel fabrication and processing facility at the SRS is slightly depleted with a ^235^U/^238^U of 0.00631 ± 0.0003. A similarly depleted signature of 0.00421 ± 0.0003 occurs in the bulk sample from the ORR. We observed enriched ^235^U/^238^U ratios in both the scute from the Mohave desert tortoise in southwestern Utah (0.00739 ± 0.00006; statistically significant from natural uranium [Wilcoxon signed-rank test, *P* < 0.05]) and the green sea turtle from Enewetak Atoll, RMI (0.00981 ± 0.0002).

**Table 1.** Uranium concentration and isotope data for all samples.

Common name	Taxon	Location	Specimen code	Date	Layer	Mass (mg)	[U] ng/g (± 5%)	^234^U/^238^U	±2 s	^235^U/^238^U	±2 s	^236^U/^238^U	±2 s
Green sea turtle	*C. mydas*	Enewetak A.	BPBM-6179	1978	Bulk	30.2	20.17	9.27E^−05^	2.61E^−06^	0.00981	0.00023	1.42E^−05^	8.38E^−07^
Mohave desert tortoise	*G. agassizii*	SW Utah	UMNH:Herp:3571	1959	Bulk	22.4	6.58	1.16E^−04^	6.03E^−06^	0.00739	0.00006	7.27E^−06^	2.18E^−06^
Sonoran desert tortoise	*G. morafkai*	BMGAFR	UNM	1999	Bulk	60.6	28.01	6.26E^−05^	1.79E^−06^	0.00728	0.00003	7.54E^−07^	7.43E^−07^
River cooter	*P. concinna*	SRS	KU-204360	1985	Bulk	26.9	23.24	6.28E^−05^	1.91E^−06^	0.00631	0.00033	5.15E^−05^	2.32E^−06^
Eastern box turtle	*T. c. carolina*	ORR	DMNH-3835	1955–1962	Bulk	32.2	115.44	3.04E^−05^	6.56E^−07^	0.00421	0.00022	3.75E^−05^	8.46E^−07^
				1955	Neonatal	18.293	60.68	2.04E^−05^	3.03E^−07^	0.00317	0.00001	3.49E^−05^	4.47E^−07^
				1956	1	13.92	17.96	5.40E^−05^	1.13E^−06^	0.00651	0.00002	4.05E^−05^	9.44E^−07^
				1957	2	26.237	14.1	4.69E^−05^	8.50E^−07^	0.00584	0.00002	3.51E^−05^	7.87E^−07^
				1958	3	18.365	28.86	6.86E^−05^	8.23E^−07^	0.00772	0.00002	4.50E^−05^	7.72E^−07^
				1959	4	15.984	16.27	4.77E^−05^	1.04E^−06^	0.00584	0.00002	4.45E^−05^	1.10E^−06^
				1960	5	15.885	19.52	4.48E^−05^	1.03E^−06^	0.0056	0.00003	3.84E^−05^	8.98E^−07^
				1961	6	30.696	14.66	4.63E^−05^	8.13E^−07^	0.00573	0.00003	3.77E^−05^	6.58E^−07^
				1962	7	15.344	11.73	4.96E^−05^	1.83E^−06^	0.00602	0.00002	3.34E^−05^	8.68E^−07^

Ratios presented are atomic ratios, not activity ratios.

Enewetak A., Enewetak Atoll, Republic of the Marshall Islands; SW Utah, southwestern Utah; BMGAFR, Barry M. Goldwater Air Force Range; SRS, the Savannah River Site; and ORR, Oak Ridge Reservation.

**Fig. 4.**
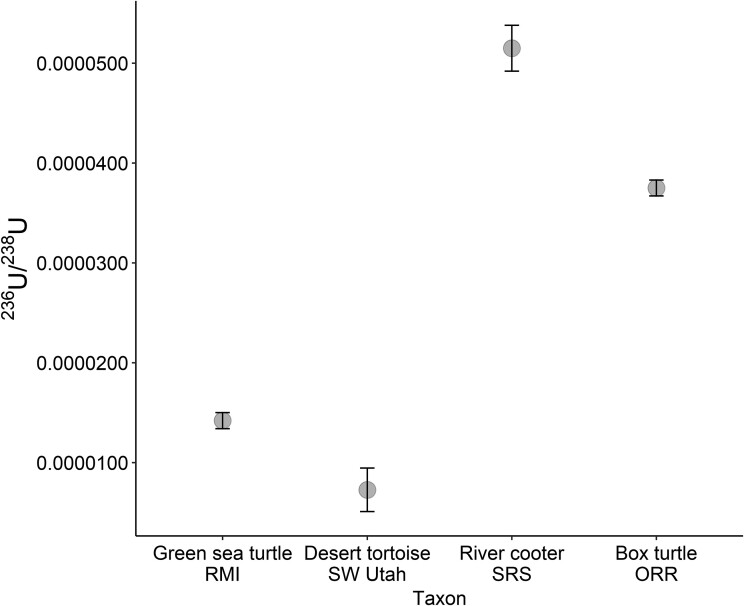
The ^236^U/^238^U isotopic ratio and error for bulk turtle scute tissue samples from this study. RMI, Enewetak Atoll, Republic of the Marshall Islands; SW Utah, southwestern Utah; SRS, the Savannah River Site; ORR, Oak Ridge Reservation.

Similar to the ^235^U/^238^U results, an anthropogenic ^236^U signature was observed in four out of the five scute samples analyzed (Fig. 4; Table 1), correlating with the deviations seen in ^235^U/^238^U. Only the Sonoran desert tortoise lacked any detectable ^236^U, but this also correlates with the natural ^235^U/^238^U we observed. The Mohave desert tortoise from southwestern Utah has a ^236^U/^238^U of 7.27 × 10^−6^, just above the instrument detection limit of 0.02 fg. The scutes from ORR, the SRS, and Enewetak Atoll had much higher levels of ^236^U with a ^236^U/^238^U of 3.75(±0.08) × 10^−5^, 5.15(±0.20) × 10^−5^, and 1.42(±0.08) × 10^−5^, respectively, all clearly above analytical detection limits.

### Temporal (annual) samples

**Fig. 5.**
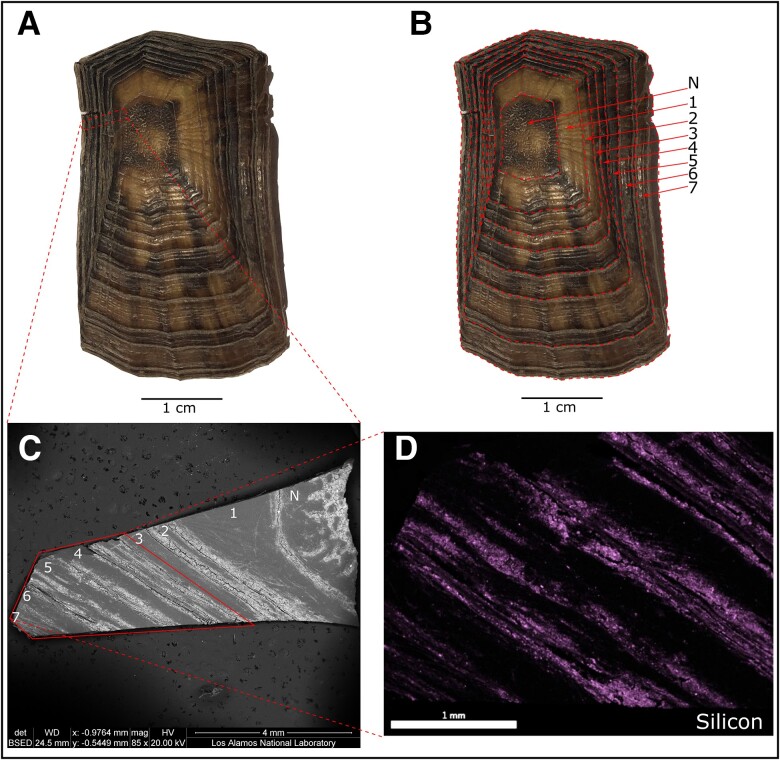
Macro and microscopic visualizations of the scute growth sequence from the eastern box turtle collected from Oak Ridge Reservation in 1962. A) the costal scute showing the location of a subsample selected for scanning electron microscope (SEM)-energy dispersive X-ray spectroscopy (EDS), B) the costal scute showing the visual location of macroscopically identifiable growth rings, C) an SEM backscatter electron image of the scute subsample showing the location of the EDS analysis, and D) an EDS analysis highlighting the presence of silicon (Si) on the scute tissue. Note that silica occurs between areas with homogenous scute growth, suggesting the presence of accumulation during the dormant-hibernation phase of the box turtles annual life cycle.

Macroscopic, scanning electron microscopy and energy dispersive X-ray spectroscopy (SEM-EDS) identified seven distinct yearly growth layers in the eastern box turtle collected in 1962 from ORR (Fig. 5). The position of major growth-ring boundaries, formed during the dormant-hibernation season (26), also corresponds to the locations where exogenous materials aggregate onto the keratin scute tissue. These exogenous materials, especially silica, provide an elemental map supporting our macro and microscopic counting of sequential layers of yearly growth in the turtle's keratin shell. Based on these results, the scute layers from this specimen age between 1955 and 1962.

**Fig. 6.**
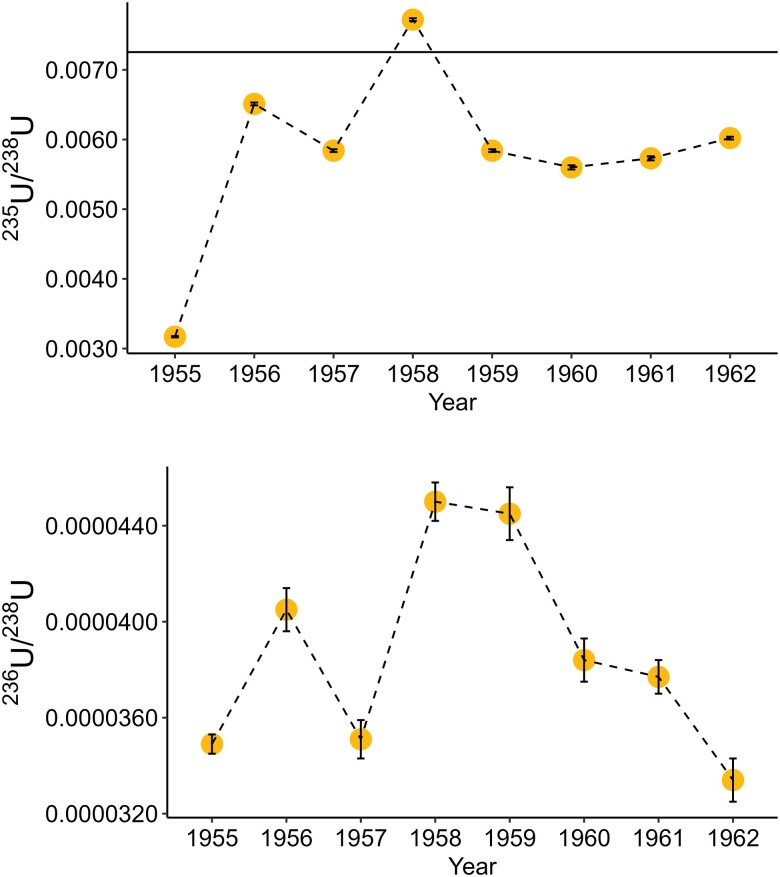
(A) ^235^U/^238^U isotopic ratios and error in the eastern box turtle from Oak Ridge Reservation and B) ^236^U/^238^U isotopic ratios and error for this same taxon. Natural uranium (0.007253–0.007256) is visible as a horizontal line in plot A.

As with the bulk scute analyzed from the SRS, there is a depleted ^235^U/^238^U isotopic signature in the box turtle from ORR. However, the degree of depletion varies over time (1955–62) with the most depleted signature in the neonatal portion (corresponding to 1955) with a ^235^U/^238^U = 0.0032 (Fig. 6). In 1956, the ^235^U/^238^U ratio increases and is only slightly depleted with a value of 0.0065, followed in 1957 with a ^235^U/^238^U value of 0.0058. In 1958, the ^235^U/^238^U ratio recorded in the scute increases slightly above natural with a ^235^U/^238^U = 0.0077. From 1959–62, the ^235^U/^238^U signature remains depleted ranging between 0.0056 and 0.0060. Similar to the bulk scute from the SRS, all eight layers of the ORR scute have measurable ^236^U with a ^236^U/^238^U ranging from 3.34 × 10^−5^ to 4.50 × 10^−5^.

## Discussion

Our data support previous studies on chelonian taxa—and numerous other organisms (3–6)—determining that these animals, in some cases significantly, bioaccumulate anthropogenic radionuclides from the environment (9–14). While these previous studies focused on radionuclide accumulation in large bulk tissue samples (or radiocarbon measurements in sea turtle scutes (15)) that primarily measure radioactivity in becquerels or elemental mass, we have demonstrated the capability to measure uranium isotopes in small amounts of shell scute keratin (<60 mg; see Table 1) at extremely sensitive levels of occurrence. Using MC-ICP-MS with detection capabilities on the order of ng/g, we now confirm the potential for analyzing scute materials as an inert reservoir for anthropogenic uranium (^235^U and ^236^U) radionuclides, even when the total amount of uranium bioaccumulated is a minute fraction of the sample. In addition, the unique age and sampling locations of the turtles in this study present the opportunity to determine whether chelonian taxa provide historical information on the characteristics of uranium contamination from individual sites and nuclear events, and for environmental monitoring programs.

We recognize the limitations present in our study, specifically that our analysis only includes individual turtles from each location and time. Furthermore, due to fragmented and mature (homogenized) scutes present in our other samples, only at the ORR was there potential to sample sequentially grown scute to obtain a diachronic record. As elaborated below, this limitation does not minimize the significance of our results as our evidence clearly associates each turtle with nuclear activities at each site during the 20th century.

Here, we discuss each individual sample in context to the environment of anthropogenic contamination that it was likely exposed to, based on historical knowledge of nuclear activities in each area. We conclude with a discussion of the wide potential for future research based on our findings.

### The Republic of the Marshall Islands (Enewetak Atoll)

There is a significant legacy nuclear signal present in a green sea turtle scute collected at Enewetak Atoll in 1978, recorded by enriched ^235^U/^238^U and a ^236^U/^238^U isotope signature. While the measured uranium concentration in the scute is low (20.17 ng/g), the measured ^235^U/^238^U of 0.00981 ± 0.0002 is approximately a factor of 1.3 times higher than natural uranium (27) (^235^U/^238^U = 0.007253–0.007256). We note that the RMI received a high degree of fallout from the testing of 67 nuclear weapons between 1946 June 30 and 1958 August 18. Fallout products at Enewetak Atoll contaminated the immediate environment, flora, and fauna, following each detonation (28), but few studies explore the signature of uranium contamination within these contexts (see summary (29)). Our results are therefore compelling, as enriched ^235^U is a known fallout product from the deployment of above ground nuclear weapons, especially from uranium-fueled devices (30). Enriched ^235^U/^238^U ratios occur in the “black plaster” surfaces (caused by the “Little Boy” weapon and subsequent “black rain” fallout) in Hiroshima, Japan (31, 32). Black rain at Hiroshima, which caused black plasters, occurred because of fires depositing significant quantities of ash into the atmosphere, which subsequently rained down (as water, ash, and radioactive fallout) onto the city. Additionally, HEU signatures occur up to 88% ^235^U in glassy fallout derived from an above ground test of a single uranium-fueled nuclear device (30).

These results are significant when placing this green sea turtle in context of its date of collection. In May 1977, one year prior to the collection of this sea turtle in 1978, cleanup activities began at Enewetak Atoll resulting in the creation of the Runit Dome containment structure. Green sea turtles are migratory, but live, forage, and nest, at Enewetak Atoll (33, 34). The presence of uranium contamination in this green turtle ∼20 years after nuclear testing ended in the RMI thus suggests the potential that cleanup activities disturbed contaminated sediments which (re)input small quantities of local fallout products into the surrounding environment. Consumption of contaminated algae or seagrass or ingestion of contaminated sediments during nesting all are potential sources of uranium for this green turtle (see Balazs et al. (35) for plutonium contamination in sea turtles from Johnston Atoll; see also Johansen et al. (12) and Rudrud et al. (34)). It is also possible that legacy contamination present in the Enewetak Atoll lagoon occurred in substantive quantities to contaminate this turtle at some point during its lifetime, regardless of potential impacts during cleanup for Runit Dome. Further research on radionuclide contamination in RMI sea turtles is required to untangle these possible sources.

### Southwestern Utah and the Nevada Test Site

A more subtle isotope signature is present in the Mohave desert tortoise collected in 1959 from the far southwestern region of Utah. It has a slightly enriched ^235^U/^238^U ratio coupled with a minor ^236^U/^238^U signal. These results are notable, however, given the distance of this location from the (formerly) Nevada Test Site (∼150 miles due east). We note that southwestern Utah did receive nuclear fallout from testing at the NNSS during the 1950s and early 1960s (36). These results suggest that this Mohave desert tortoise accumulated a uranium fallout signature at a considerable distance from a known source of testing. A Sonoran desert tortoise collected from the Barry M. Goldwater Air Force Range in southwestern Arizona acts as a control for our measurements as it was unlikely exposed to any significant input of anthropogenic uranium, with exception of global fallout. This Sonoran desert tortoise has a natural uranium ^235^U/^238^U ratio with no associated ^236^U/^238^U and provides further confidence in the deviations we report from southwestern Utah and elsewhere.

### The Savannah River Site

The uranium isotope composition recorded in the river cooter from the SRS confirms previous research that identified bioaccumulation of radionuclides in chelonians at the site (14), particularly within aquatic systems and/or retaining ponds for contaminated wastewater. Interestingly, we observe a plausible link between the DU signature in this turtle scute and a known contamination event at the SRS. Although the river cooter specimen only represents ca. one year (∼1985) of environmental data, documented contamination records from the SRS indicate that in 1984, there was a large release of uranium into the environment (18). This release occurred at a chemical separation facility that involved depleted uranium activities. A leak in the cooling coils at this facility led to releases of uranium in the 1950s and 1984 specifically. Unfortunately, an exhaustive records search failed to indicate the exact location of collection within the SRS, and thus this link is not certain, but this river cooter has a clear depleted ^235^U/^238^U signal with an associated ^236^U/^238^U ratio from ca. 1985.

### Oak Ridge Reservation

A comparable DU signature also occurred in the scute samples of the eastern box turtle collected from ORR in 1962 which matches known environmental release data (23). Using this sample, we demonstrate the ability to measure uranium isotope signatures within individual, sequentially grown, scute layers. Our analysis of the scute layers, coupled with the known date/year of collection, provides a time constrained growth sequence from 1955–62 revealing variation in both uranium concentration and isotope signatures from layer to layer, or year to year. This suggests that the turtle experienced and passively recorded variation in the degree and signal of uranium contamination in its environment over the course of its life at ORR (see Seltzer and Berry (37) for a comparable study on heavy metal bioaccumulation in tortoises). Surprisingly, the neonatal scute, which develops within the egg during embryogenesis (see Cherepanov (38)), had both the largest concentration of uranium and the most depleted ^235^U/^238^U signal within our intra-scute samples. This is significant as the elemental composition of the box turtle's scute at this phase of life relates directly to the individual's egg-laying mother. Embryos obtain elemental constituents from the parent during egg formation—these elements are drawn from whole-body bulk tissues and reflect long periods of deposition (i.e. not routed through a single feeding event)—and previous research indicates that contamination and pollutants are passed through eggshells to offspring ((39); see related (40)). We therefore suspect that the neonatal scute signature records the contamination experienced by the mother turtle over a period of her lifetime.

**Fig. 7.**
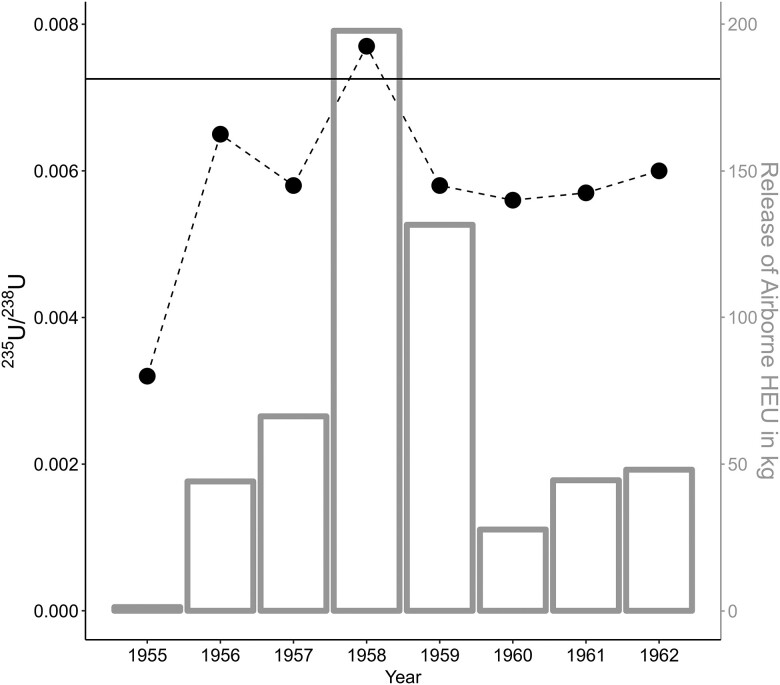
Historic uranium airborne release data (in kg) from Oak Ridge Reservation (right axis) represented by bars plotted with the ^235^U/^238^U isotopic ratio (left axis) points from the box turtle sample dating between 1955 and 1962. Natural uranium (0.007253–0.007256) is visible as a horizontal line.

Although we do not have access to the mother of this turtle, the presence of anthropogenic uranium within this turtle's neonatal scute suggests that nuclear contamination passed between at least two generations of turtles at ORR during the mid-20th century. In addition, we note that the highly depleted uranium isotope signature matches historical records that show pre-1955 uranium waste streams at ORR were of a heavily depleted nature. For example, documented releases of wastewater from ORR's Y-12 Complex—originally constructed during the Manhattan Project to enrich uranium during the 1940s–1960s—resulted in depleted uranium contamination throughout the environment (23, 41, 42). This liquid contamination, depleted in ^235^U/^238^U, settled in containment ponds and throughout nearby streams and creeks (e.g. Bear Creek and White Oak Creek for the X-10 Complex), the possible habitat of our box turtle. Variation in the isotope signature recorded in these successive layers indicates that the turtle continued to record environmental uranium contamination over time. Indeed, recorded data from the East Fork Creek highlight a consistent concentration of uranium (measured in mg/L) within this waterway during our study years (Fig. 7; 1955–62). Except for one year (1958), the recorded signatures correlate with contamination from a DU source. The isotope signature recorded from 1958 is distinct as it comprises a slightly enriched ratio. Given the overall depleted signature recorded within the other layers, we believe that this layer records a separate input of HEU into an environment already contaminated with DU. Our data from 1958 correlate with historical records showing a large release of enriched ^235^U/^238^U at ORR and within the Scarboro community (adjacent to Y-12) specifically in 1958.

### Conclusions and future studies

When we consider the legacies of 20^th^-century nuclear deployment, testing, and production, we must now also consider chelonians and their lived experience in areas where these activities occurred. Our results confirm modeled expectations (43) showing that chelonians should bioaccumulate and retain anthropogenic radionuclides across their shell scute keratin. These animals are thus uniquely positioned to record information about human activities in nuclear landscapes over the long-term. We anticipate that combining analyses of historically collected and modern specimens will significantly expand our environmental monitoring abilities as they relate to ongoing nuclear contamination questions.

For example, given our limited sample size, we anticipate that future studies will focus on expanding the types of chelonians, and chelonian scute sequences, analyzed for anthropogenic radionuclides (including other nuclear elements). Turtles inhabiting locations with significant nuclear deployment histories (e.g. Japanese pond turtles [*Mauremys japonica*]), nuclear test histories (e.g. Kazakhstan steppe tortoise [*Testudo* (*Agrionemys*) *horsfieldii*]), or nuclear processing, production, and accident histories (e.g. Ukrainian pond turtles [*Emys orbicularis*]) will undoubtedly clarify the extent to which these reptiles bioaccumulate and reflect anthropogenic contamination in the environment. We anticipate that our green sea turtle results may influence future sea turtle-based environmental monitoring and tracking of contaminates at Runit Dome in the RMI, and potentially long-term releases of radioactive water from the Fukushima Daiichi reactors, into the Pacific Ocean.

Our analysis also suggests that sequentially grown tissues from a variety of organisms may provide reliable data for identifying nuclear events. Molluscan tissues (e.g. bivalve growth layers (44)), corals (45), certain plant tissues (e.g. cactus spines (46)), shark and/or fish eye lenses and otoliths (47), bird feathers (48), select mammalian teeth (49), and more all have the unique potential of providing time series isotopic records. Combining analysis of a variety of organisms from nuclear sites in conjunction with localized sediments, plants, and water will also help inform the exact pathway of contamination for specific samples. As plutonium-239 and -240, cesium-137, and strontium-90 anthropogenic radionuclides also accumulate in tissues, we expect future research on these elements and isotopes to further our understanding of specific nuclear events. Our data reveal that chelonian scutes record signatures that are significant and characteristic of the anthropogenic nuclear input from a specific region and time, providing the ability to reconstruct legacy environmental contamination records from across a wide range of uranium contamination events. Chelonian scute now becomes a clear proxy tissue for examining temporally controlled samples and deciphering historic nuclear inputs into the environment.

## Materials and methods

### Samples

We obtained chelonian scute samples from natural history collections for this analysis. Prior to obtaining each sample, we received Los Alamos National Laboratory Institutional Biosafety Committee approval.

### Scute layer counting

To age the eastern box turtle specimen, we used macro and microscopic techniques. Four of our five chelonian samples only included a single bulk tissue specimen for this analysis, therefore, we accept the date at death (or collection) as the calendrical date for that sample (i.e. samples are homogenized and include all scute layers). Turtles grow scute layers in a variety of forms, including sequentially in overlapping concentric rings, or by growth of a new scute layer while retaining former layers in sequence (50). River cooter turtles shed scutes after a period of growth, and thus the exterior scute is a bulk sample reflective of that calendar year. In only one sample, the eastern box turtle, did we macroscopically count the number of rings present and microscopically count rings using a scanning electron microscope coupled with EDS. These combined techniques resulted in analysis of: (i) a green sea turtle (ca. 1978), (ii) a Mohave desert tortoise (ca. 1959), (iii) a Sonoran desert tortoise (ca. 1999), (iv) a river cooter (ca. 1985), and (v) an eastern box turtle (ca. 1955–62).

### SEM-EDS

Samples were mounted on aluminum stubs with double-sided carbon tape, and carbon coated using a Cressington 108carbon/A system. Images and analyses were collected with an FEI Quanta 200F field emission SEM coupled with an EDAX Octane Silicon Drift Detector for EDS analysis. Images were collected using both secondary electron and backscatter electron detection for textural and Z-contrast information. Images and EDS point analysis spectra were collected with a 20-kV accelerating voltage and an FEI spot size setting of 4 (moderate spot size optimized for both imaging and EDS).

EDS point analyses were collected using a working distance of 10 mm, a duration of 2 min, and an amp time of 7.68 μs. EDS element composition data were processed using EDAX Team software and employed the PeBaZAF (Peak to Background ZAF—chosen due to sample surface roughness) Smart Quant method, using an assumed 50-nm carbon coat. Elemental compositions are standardless and normalized, and are therefore semi-quantitative. EDS element maps were collected with a 20-kV accelerating voltage with a larger FEI spot size setting of 5. EDAX Team software parameters were as follows: an amp time of 0.12 μs, a dwell time of 200 μs, a resolution of 1280 × 1024 pixels, and 256 frames, for a resulting map run time of 13.5 h.

### MC-ICP-MS and QQQ-ICP-MS

Samples were dissolved in a class 100 laminar flow hood using a hot plate, pre-cleaned 15-mL PFA teflon beakers (Savillex, USA), Optima grade reagents (Fisher Scientific, USA) and 18.2-MΩ H_2_O. The high organic content of the samples required a two-step dissolution procedure. Samples were first dissolved at ∼120°C for 12 h in 5-mL concentrated HNO_3_. The samples were then evaporated to dryness, reconstituted with a 5-mL mixture of concentrated HNO_3_, and 30% H_2_O_2_ (4:1 v/v) and placed on the hotplate for a further 24 h. This second dissolution step was found to be critical in eliminating any remaining organics from the sample, which have the potential to interfere with mass spectrometry. The samples were then evaporated to dryness and reconstituted in 3N HNO_3_ prior to chemical separation of uranium. Uranium separation was completed using a TRU spec (Eichrom Industries, USA) method from Reinhard et al. (51), which has been shown to work well with highly organic samples. Uranium blanks for the combined dissolution and uranium separation procedure totaled <10-pg and represents only a minor component of the total uranium measured in each sample.

We measured the isotopic composition of the scute samples using a Thermo Neptune Plus MC-ICP-MS at LANL. The dissolved and processed samples were redissolved in a 2% HNO_3_ solution and introduced into the MC-ICP-MS using a Cetac Aridus 3 desolvating nebulizer system and a 100-μL/min Savillex nebulizer. The samples were measured using a combination of ion counters (for ^234^U, ^235^U, and ^236^U) and a Faraday detector (for ^238^U). The baseline and gain for the Faraday detector and dark noise for the ion counters were determined at the start of the analytical session. The instrument mass bias and ion counter yields were determined by measuring a solution of U010 throughout the analytical session bracketing the samples and quality control (QC) standards. We used two QC standards to verify the accuracy of the mass bias correction and ion counter yields as follows: CRM U005A (a DU standard containing ^234^U and ^236^U) and NBS SRM 960 (a natural U standard). The QC standards were run at concentrations to match the signal intensities measured in the samples.

Uranium concentration measurements were made using an Agilent 8900 triple quadrupole ICP-MS (QQQ-ICP-MS) at LANL. The first quadrupole was arranged as an ion guide, and no gas was introduced to the collision/reaction cell. The samples were introduced into the QQQ-ICP-MS using a PFA Microflow (200) nebulizer and a double pass quartz spray chamber. An acid blank and NBS SRM 960 uranium standards were measured throughout the analytical session to evaluate sample washout and analytical drift. Uranium concentrations were determined by building an eight-point calibration curve, including an acid matrix blank, and increasing concentrations of SRM 960 solutions of 0.01, 0.12, 0.26, 0.50, 0.72, 1.07, and 2.96 ng/g. The concentration of the samples was calculated by creating a linear calibration curve and applying to the measured intensities of the samples.

All our data and supplemental coding script are available through an open-access repository at osf.io (52).

## Data Availability

All associated data for this manuscript is archived at osf.io: https://doi.org/10.17605/OSF.IO/JMZKG
